# Label-free photothermal optical coherence microscopy to locate desired regions of interest in multiphoton imaging of volumetric specimens

**DOI:** 10.1038/s41598-023-30524-z

**Published:** 2023-03-03

**Authors:** Naresh Kumar Ravichandran, Hwan Hur, Hyemi Kim, Sangwon Hyun, Ji Yong Bae, Dong Uk Kim, I Jong Kim, Ki-Hwan Nam, Ki Soo Chang, Kye-Sung Lee

**Affiliations:** Center for Scientific Instrumentation, Korea Basic Science Institute, 169-148 Gwahak-ro Yuseong-gu, Daejeon, 34133 Republic of Korea

**Keywords:** Interference microscopy, Imaging and sensing, Biophotonics

## Abstract

Biochip-based research is currently evolving into a three-dimensional and large-scale basis similar to the in vivo microenvironment. For the long-term live and high-resolution imaging in these specimens, nonlinear microscopy capable of label-free and multiscale imaging is becoming increasingly important. Combination with non-destructive contrast imaging will be useful for effectively locating regions of interest (ROI) in large specimens and consequently minimizing photodamage. In this study, a label-free photothermal optical coherence microscopy (OCM) serves as a new approach to locate the desired ROI within biological samples which are under investigation by multiphoton microscopy (MPM). The weak photothermal perturbation in sample by the MPM laser with reduced power was detected at the endogenous photothermal particles within the ROI using the highly sensitive phase-differentiated photothermal (PD–PT) OCM. By monitoring the temporal change of the photothermal response signal of the PD–PT OCM, the hotspot generated within the sample focused by the MPM laser was located on the ROI. Combined with automated sample movement in the x–y axis, the focal plane of MPM could be effectively navigated to the desired portion of a volumetric sample for high-resolution targeted MPM imaging. We demonstrated the feasibility of the proposed method in second harmonic generation microscopy using two phantom samples and a biological sample, a fixed insect on microscope slide, with dimensions of 4 mm wide, 4 mm long, and 1 mm thick.

## Introduction

Multiphoton microscopy (MPM) allows for high-resolution analyses in various biological research fields. In recent years, the use of MPM has continued to gain attraction in biomedical imaging, especially in areas of in vivo and live deep neuron imaging in small animals^1–5^, early detection of tumors and its characterization^6–9^, vascular network and organoid imaging in its microenvironment in bio-chips^10–12^. There have been many approaches to increase the imaging depth, larger field of view (FOV), and shorten the volumetric scanning time by adopting appropriate fluorophores^13^, adaptive optics^14^, multi-beam and multi-focal mechanisms^15,16^, in addition to other similar modifications in the optical setup which has given variations of MPM imaging systems adoptable as per imaging needs and application scenarios ^5,16–21^.

Photodamage and photobleaching are critical factors to consider in multiphoton imaging. Prolonged illumination accompanied with high laser power necessary for label-free multiphoton imaging, there is photomechanism of tissue damage that results in enhanced fluorescence causing cell death, which is widely referred to as photodamage ^22–24^. To avoid photodamage occurrence in living cells, care must be taken to ensure the imaging parameters are well below the photodamage threshold like peak intensity, repetition rate, and prolonged exposure (dwell) times^24^. Most of these parameters can be controlled by the source and detection systems. Multiple research groups studied and proposed various approaches to suppress the effects of photobleaching and photodamage. The simplest and most widely adopted method is to reduce the total illumination power of a light source^4,25,26^. However, this, in turn, reduces the overall sensitivity of the system due to the reduced signal-to-noise ratio. Alternative approaches reported to mitigate this effect involve a controlled light-exposure method that spatially controls the light exposed onto the sample, the use of a passive pulse splitter that redistributes the illumination laser pulse into sub-pulses with equal energies, and a fast optical scanning mechanism that reduces photodamage by controlling the illumination and collection of emission from specimens^27–30^. In addition, using the light-sheet illumination method, only the focal plane of the detection objective is effectively illuminated^31^. Although these methods are useful for reducing photodamage and photobleaching effects, long-term high-power illumination is required when searching for region of interest (ROI) in large samples with small FOVs and long volumetric scanning times, thus resulting in photodamage and photobleaching.

In recent decades, multiple research groups reported the advantages of incorporating both MPM and optical coherence tomography (OCT) as a single multimodal imaging platform, where their advantages compensate for each other modalities limitations^32–35^. In particular, MPM and OCT imaging techniques have different characteristics in terms of resolution, FOV, imaging depth, and imaging speed. For example, MPM uses a nonlinear mechanism that requires a tightly focused volume of the laser beam in the sample with a high numerical aperture (NA) objective that produces high lateral and axial resolutions but a small FOV. Moreover, OCT is a linear imaging system that provides increased flexibility in choosing the resolution and FOV that suits your application. In terms of imaging depth, MPM typically provides imaging depths of several hundred micrometers in biological samples where high scattering and aberrations exist, whereas OCT utilizes the higher penetrating power of near-infrared light sources to achieve imaging depths of 1–2 mm in biological samples. Generating volumetric images of samples using an MPM system requires the use of a z-axis stage with two-dimensional scanning in the transverse direction to move the sample axially. In contrast, OCT can acquire volumetric images using only raster x–y scanning without moving the sample axially. The combined MPM-OCT system can serve as an effective multiscale-imaging platform for biological investigations by the complementary use of the superiority of each system in imaging.

Since the introduction of OCT, various research groups investigated and utilized the different functional properties of the laser source and detection mechanism to establish new methods with respect to imaging requirements. Several examples include Doppler OCT for flow measurements in samples^36^, polarization-sensitive OCT to study birefringence properties in the sample^37,38^, optical coherence elastography to measure the stress and tensile strength of structures within samples^37,38^, spectroscopic OCT to investigate the wavelength-dependent absorption and scattering of light of structures in samples^37,38^, and photothermal OCT (PT-OCT) to analyze thermal fluctuations within samples^39–41^. Among these technologies, PT-OCT has attracted significant research attention for the evaluation of the thermal fluctuations of samples and the resulting changes in their physical and optical properties. In PT-OCT systems, nanoparticles, photothermal responsive exogenous contrast agents, and absorbers have been utilized to generate and measure the photothermal effects within the sample^42–45^. To date, studies using endogenous contrast agents instead of external contrast agents have been limited due to the lack of usefulness and the difficulty in measuring weak photothermal response signals. Milner et al. proposed a differential phase measurement for the depth-resolved detection of photothermal response in tissue, without the use of external contrast or absorber material^46,47^. Although the proposed photothermal response detection technique can be applied to samples without the need for contrast or absorbers, it requires multiple dedicated detection channels and a rapid scanning optical delay to match the two different interferometric paths in the sample scanning introduced by the birefringent material. Furthermore, the required illumination laser power is typically 80–100 mW for biological samples, to measure the photothermal response with reasonable signal-to-noise ratio^47^. Recent advances in PT-OCT techniques have enabled effective photothermal response measurements using endogenous contrast using a single detection channel and with reduced laser illumination power of 4–10 mW^39,48–50^.

This study serves as a precursor to the direction of a new ROI location mechanism along three axes within the sample volume to reduce the overall photodamage and photobleaching effects and improve user convenience in multiphoton imaging of volumetric samples. The desired ROIs were selected within the complete observable depth and lateral range of Optical coherence Microscopy (OCM). The changes in the optical properties of the sample due to the photothermal effects illuminated by the MPM laser source were measured at the endogenous photothermal particles within the ROI using the highly sensitive phase-differentiated photothermal (PD–PT) OCM. By monitoring the temporal change of the photothermal response signal of the PD–PT OCM, the focus position of the excitation laser (hotspot) was placed on the ROI. Combined with automated sample movement in the x–y axis, the focal plane of MPM could be effectively navigated to the desired portion of a volumetric sample for high-resolution targeted MPM imaging. The ROI navigation was attained using a low-powered (lower than conventional power by a factor of less than 10) MPM laser source. The PD–PT OCM guiding method can be implemented for any nonlinear imaging technique that requires high-power laser illumination, which leads to photobleaching and photodamage in samples, e.g., two-photon excited fluorescence and coherent anti-Stokes Raman scattering microscopy.

## Theory

When a high-energy laser beam irradiates a sample, the internal temperature and optical properties of the internal sample structure changes^51^. In the OCT imaging technique, the detected interference signal in the Fourier domain OCT can be expressed as Eq. (1) in complex form:

1 $$I\left(z,t\right)\propto 2\sqrt{{I}_{R}{I}_{S}}\mathrm{exp}\left(i\Phi \left(z,t\right)\right)$$

where z is the path length difference between the reference mirror and interface within the sample. The ${I}_{R}$ and ${I}_{S}$ are the backreflected light intensities from the reference mirror and the sample interface, respectively. The $\Phi \left(z,t\right)$ is the phase term of the interference signal, which can be represented as an integral function of the depth-dependent refractive index in space at a certain point in time, as expressed by Eq. (2):

2 $$\Phi \left(z,t\right)=\frac{2\omega }{c}{\int }_{0}^{z}n\left(z,t\right) dz$$

where *ω* is the central angular frequency of the light source, and *c* is the speed of light in vacuum. The sample is assumed to be located on the positive side in the space domain, and $n\left(z,t\right)$ represents the depth and time-dependent refractive index within the sample. When a temperature change occurs within the sample, the thermal response optically observed in the sample is a phase change due to the thermoelastic and thermorefractive effects. Thus, Eq. (2) can be extended considering the thermal responses, as expressed by Eq. (3) (Ref.^47^).

3 $$\Phi \left(z,t\right)=\frac{2\omega }{c}{\int }_{0}^{z}\left[n\left(z\right)+\left(\frac{dn}{dT}\right)\Delta T\left(z,t\right)\right]\cdot \left[1+\beta (z,T)\Delta T(z,t)\right] dz$$

where *dn*/*dT* represents the change in the refractive index with temperature, $\Delta T\left(z,t\right)$ represents the temperature change in the sample tissues, and $\beta$ is the thermal expansion coefficient^47^. To measure the phase changes in the sample due to the thermal response when illuminated by the MPM laser source, complex interference signals were obtained as digitized values with PD–PT OCM. The obtained phase term is the accumulated phase along the depth. Therefore, it first requires differentiation to resolve the accumulated phase^46,47,49^, as expressed by Eq. (4).

4 $$\Delta _{z} \Phi \left( {m,n} \right) = \left[ {{\text{F}}\left( {m + 1,n} \right) - {\text{F}}\left( {m,n} \right)} \right]$$

where $m$ is the depth index of A-scans, and $n$ denotes the designated sequential incremental indexes from the first to the last A-scan signal with time interval $\Delta t$. The total number *N* of A-scans was obtained for the same lateral position on the sample to increase the thermal response sensitivity. It takes a time period of *T* which equals $\left(N-1\right)$ times the interval $\Delta t$. The *N* of A-scans is referred to as a frame. Successive A-scans are subtracted to measure the phase change over time, as expressed by Eq. (5).

5 $${\Delta }_{t}{\Delta }_{z}\Phi \left(m,n\right)=\left[{\Delta }_{z}\Phi \left(m,n+1\right)-{\Delta }_{z}\Phi \left(m,n\right)\right]+\varphi (m,n)$$

where $\varphi (m,n)$ is the random phase noise generated in the system within the time interval $\Delta t$ for the spatial inde× *m* and temporal index *n*. In this study, 801 A-scans were obtained for the time period to achieve 800 differentiated A-scans. The differentiated phases beyond the range of −π and π were wrapped with −2π to avoid possible errors near the bounds –π and π. A specific depth $d$ of interest was selected among the depths of the A-scans, and 800 differential phase values at the depth were achieved. *d* corresponds to the product of ${m}_{s}$ and $\Delta z$, where ${m}_{s}$ is the selected depth index and $\Delta z$ is the depth interval. We averaged those differential phase values into an effective phase difference that occurred within the time period to suppress random phase errors, thus improving the thermal response detection sensitivity of the system. Phase noise mainly results from ambient thermal changes and mechanical vibrations in the system. The averaged differential phase ${\Delta \Phi }_{avg}(d)$ was then calculated using Eq. (6).

6 $${\Delta \Phi }_{avg}(d)=\frac{\sum_{n=1}^{N}\Delta \Phi ({m}_{s},n)}{N}$$

where $\Delta ={\Delta }_{t}{\Delta }_{z}$ and $\Delta \Phi ({m}_{s},n)$ denote the differential phase values at a specific depth *d* in the ${n}$th differentiated A-scan signal, respectively. This process was sequentially repeated for the desired number of times $Fn$ (frame number). In here, the term frame is not to be confused with the conventional denotation for B-scan, which is usually used in OCT imaging. The $Fn$ is the accumulated 801 A-scans from a single point. The term "frame" is used here to have the readers contemplate and correlate the sense of time it takes to have a PD–PT-OCM signal when being able to relate to a B-scan to understand the high-speed measurement capability of the proposed method. To perturb the photothermal response in the sample and generate the resultant phase change with the MPM laser source, a mechanical shutter was incorporated in the MPM illumination beam path, and was then controlled remotely to open and close at specified times within the duration of $Fn$, as per-requisite. Throughout this study, the maximum value used for $Fn$ was 50 unless mentioned otherwise. The measurement of 50 frames is referred to as the reading of the photothermal response to the perturbation and corresponds to an illumination cycle. The shutter was set to open and close near the center of each illumination cycle. This was to ensure that there was sufficient time for the sample to cool down to its natural temperature. The implementation of the shutter action for a single reading of the photothermal response and typical phase change over time is shown in Fig. 1.

**Figure 1:**
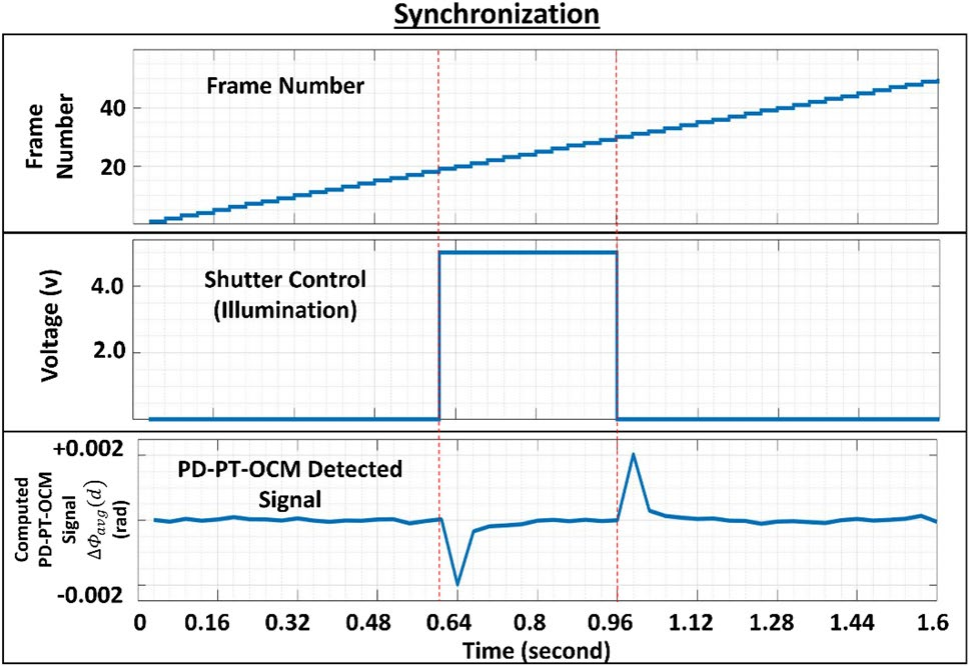
Relationship between frame number, shutter action, and photothermal response during an illumination cycle.

**Figure 2:**
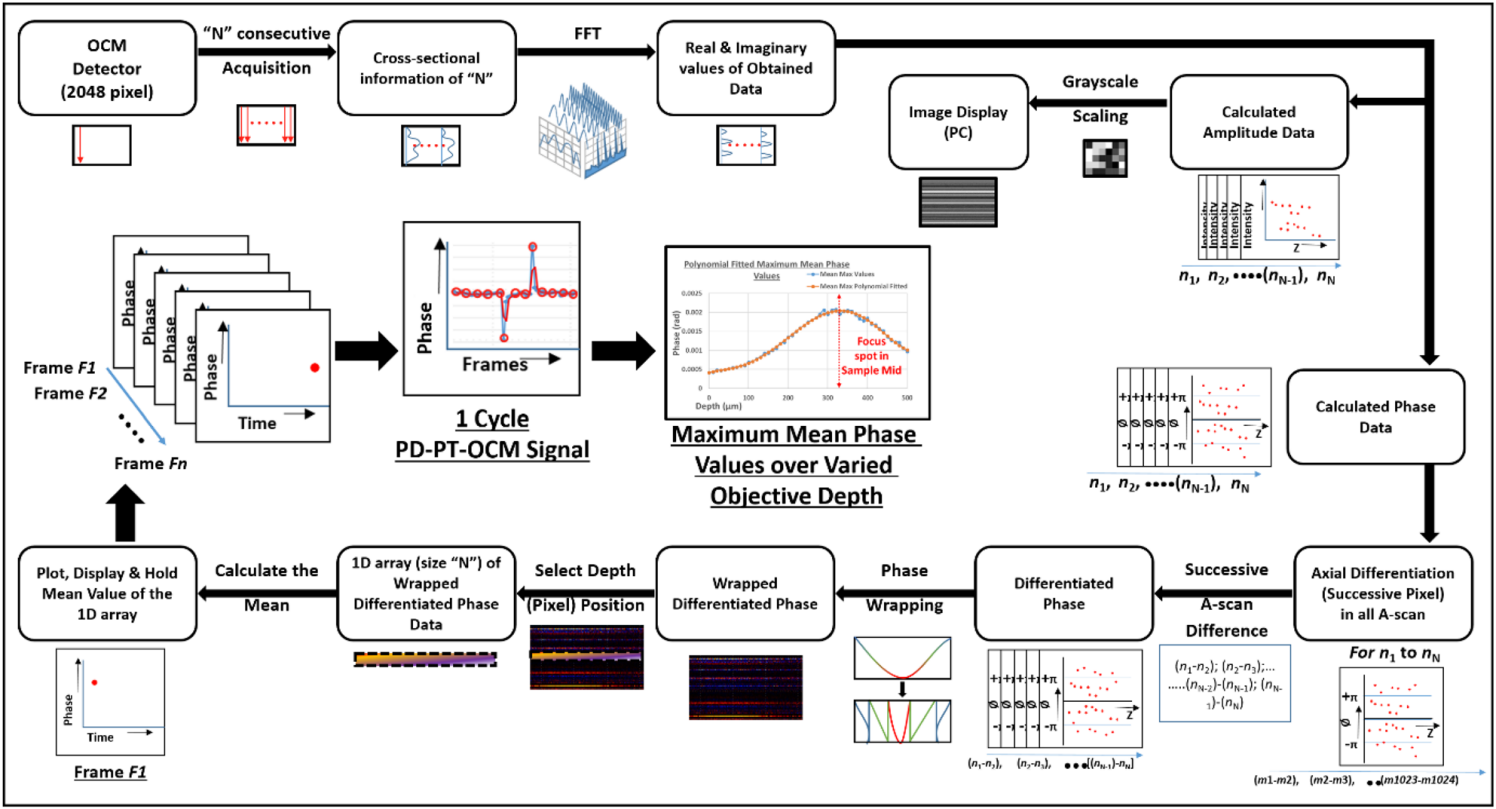
Workflow for photothermal response detection with the label-free PD–PT OCM. Step-by-step figurative description of the process involved in calculating the photothermal signal using the PD–PT OCM.

Optical endogenous absorption agents such as lipids, water, melanin, and hemoglobin etc. are widely present in biological tissues^52^. After selecting an ROI within the volumetric OCM image, an absorption agent within the ROI that exhibited an appropriate photothermal response were selected as an observation point for the photothermal response. The closer the focal position of the excitation light of the MPM to the observation point, the greater photothermal response. We measured multiple photothermal responses while varying the distance between the observation point and focal position of the illumination beam, to determine the point at which cthe photothermal response was maximized. It should be noted that the focal plane of the MPM excitation light was placed on the ROI, and the MPM image could be obtained immediately without wandering around to find the ROI. A schematic representation of the aforementioned overall workflow is shown in Fig. 2. Combined with automated sample movement in the x–y axis, the depth localization search of the ROI has been extended to be explored in three dimensions.

## Results

Two different phantom samples were fabricated and used in this experimental study to demonstrate and characterize the proposed PD–PT OCM-guided MPM. The first was a simple phantom sample used to evaluate the concept and analyze the detected photothermal signal. An additional phantom sample with a multilayered structure was used to validate the robustness of the proposed system applicability to a complex sample such as the blood vessel network of tissue structures. Finally, we applied it to an insect (*Ixodes dammini*) sample with dimensions of 4 × 4 × 1 mm, embedded in a microscope slide, to evaluate the practical applicability of the system for biological samples. Detailed information on the samples used is provided in the “Materials and methods” section.

### Proof of concept using a simple phantom sample

**Figure 3:**
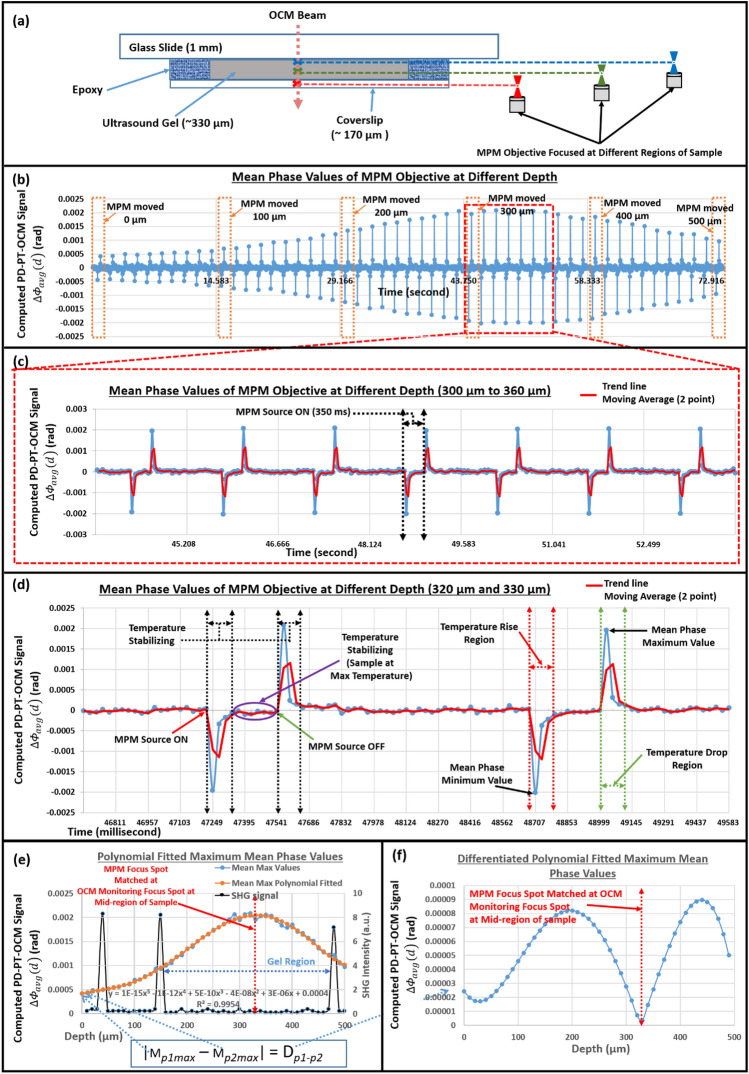
PD–PT OCM signal analysis using a simple phantom sample. (**a**) is the figurative description of MPM focus position movement over varied depth. (**b**) is the complete photothermal response observed in the simple phantom sample within a depth range of 500 µm. (**c**) is the enlarged graph of the photothermal response observed in the middle region of the sample. (**d**) is the peak photothermal response observable within the sample. (**e**) is the maximum mean phase values plotted against the depth range of 500 µm in the sample. (**f**) is the graph plotted from the absolute difference between successive values in (**b**). Figure (**a**) is not drawn to scale.

A simple phantom sample (ultrasound gel) was used to characterize and analyze the photothermal response using the proposed method. The experimental protocol was similar for all experiments conducted in this study (refer to the “Materials and methods” section for the *Simple and complex network phantom sample fabrication and experimental protocol*). The photothermal response was measured as shown in Fig. 3. Backscattering particles, such as air bubbles or dust, located in the middle region of the gel medium were selected in the cross-sectional OCM image as an observation point for the photothermal response. The focal position of the MPM objective was moved up along the optical axis of the OCM, from under the coverslip and across the ultrasonic gel area of the phantom sample, as shown in Fig. 3a. The photothermal response was measured for each 10 µm movement of the MPM objective up to 500 µm of the total travel distance corresponding to 50 readings. The total time required for 51 readings was ~ 73 s. The time spent moving manual translations was not included. As shown in Fig. 3b, the photothermal response signal at intervals of 100 µm from the total readings is indicated by the dotted rectangular box area. It can be seen that the maximum photothermal response detected at each reading exhibited a steady increase and decrease as the MPM objective focus position approached and moved away from the mid-region of the sample, respectively. As expected, the photothermal response was maximized when it passed through the middle region of the gel. Figure 3c,d are the zoomed-in graphs shown in Fig. 3b, which indicates the photothermal response signals observed when the focal point of the MPM objective was near the middle of the gel region and the detailed characteristics of the photothermal response, respectively. As can be seen throughout Fig. 3b-d, the detected photothermal response at all readings exhibited two peaks representing the phase difference change corresponding to the maximum temperature perturbation. The temperature in the sample changed most rapidly right after when the shutter was open and closed. When the shutter was open, the MPM laser beam was focused on the sample, thus inducing photothermal effects due to changes in the internal temperature of the sample, which was considered as the first temperature perturbation (temperature rise) region, as shown in Fig. 3c. When the temperature reached its maximum and stabilized, the change in phase difference returned to a value close to that at the reference temperature in the absence of illumination. When the shutter closed, the accumulated thermal energy in the sample began to decrease. In other words, when the cause of the increase in temperature (MPM laser beam) is no longer exposed to the sample the overall sample temperature to decrease and settles to its initial natural temperature. This can be seen as the second temperature perturbation region (temperature decrease), as shown in Fig. 3d.

To further analyze the amount of the photothermal response according to the change of the focal position of the objective lens, the maximum values in the photothermal signals are plotted with respect to different focal positions of the MPM objective in depth as shown in Fig. 3e. This allows for the visualization of the overall thermal response of the sample. The red dashed line in Fig. 3e indicates the selected observation point of the OCM for the photothermal response, given that the MPM objective was translated throughout the sample depth. The three intensity peaks (black) are the detected SHG signals corresponding to the first surface of the coverslip, second surface of the coverslip, and first surface of the glass slide, respectively. The observation point for the photothermal response coincides with the middle of the gel area between the two SHG signals representing the second surface of the coverslip and the first surface of the glass slide. By applying fifth-order polynomial fitting (to compensate for induced random phase noise) to the photothermal signal curve, a smooth transitional trend of the curve was obtained. Additionally, a plot representing the absolute difference between successive values after polynomial fitting is shown in Fig. 3f. This allows for the clear visualization of the instance at which the focus of the MPM objective coincided with the fixed observation point.

### Validation of robustness of ROI selection for nonlinear imaging using PD–PT OCM

**Figure 4:**
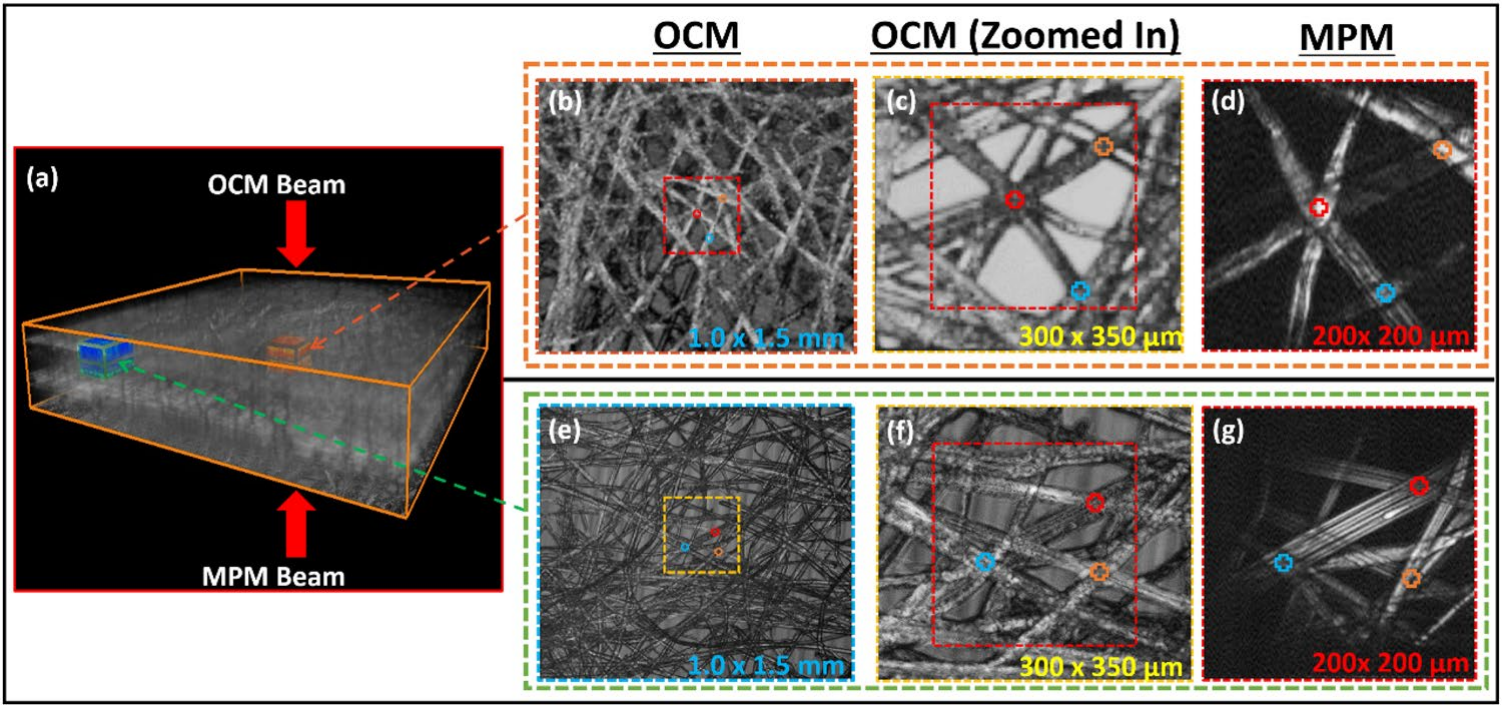
Evaluating the robustness of the PD–PT OCM-guided MPM with complex network phantom sample. (**a**) is the 3D volumetric OCM image of the sample. (**b**, **c**, **e**, **f**) are OCM *en face* images of the complex network phantom sample. (**c**, **f**) are the enlarged regions indicated by dashed squares in (**b**) and (**e**), respectively. (**d**, **g**) are the stacked SHG *en face* images of the same location scanned in (**c**) and (**f**). Additionally, in (**a**), the rectangular boxes in orange and blue indicate the stacking depth range for SHG images at the initial and moved ROIs at the desired locations, respectively.

To evaluate the ROI selection for nonlinear imaging using PD–PT OCM, large-area multilayered composite phantom samples that mimic the tissue structure of blood vessels or neuronal networks were used in the experiment. The sample contained a multilayered lens cleaning tissue with a thickness of approximately ~ 300 µm that was immersed in distilled water (refer to “Materials and methods” section for further details). It is utilized to validate the effectiveness of the ROI selection method using the real-time cross-sectional and *en face* imaging capabilities of OCM, which has a wider FOV than the limited FOV of MPM. This is shown in Fig. 4a-c,e,f. Figure 4a presents the volumetric three-dimensional (3D) OCM image of the sample with an FOV of 3.0 × 3.5 × 0.3 mm along the horizontal, vertical, and depth axes. The 3D volume image is highlighted by two rectangular ROI regions: the initial ROI (orange) and the moved ROI (blue) at a desired locations for MPM imaging. Figure 4b,e present the *en face* OCM images with an FOV of 1.0 × 1.5 mm obtained at the ROI regions. Figure 4c,f present enlarged images of the ROIs shown within the rectangular blue and yellow box areas in Fig. 4b,e, respectively, which were used for comparison with SHG images. The first observation point within the initial ROI was selected at a depth of 100 μm, where it was close to the coverslip. The focal plane of the MPM objective was positioned using the ROI selection mechanism. Figure 4e-g were obtained after moving the sample to 1000 μm in the lateral directions of X and Y, respectively, and 100 μm in the depth direction towards the glass slide (Z-axis); to simulate the navigation of different ROIs (refer to “Materials and methods” section for further details). Before sample movement, the selected region of the tissue structure for MPM imaging was close to the coverslip; and after sample movement, the region of tissue structures to be imaged was relocated closer to the first surface of the glass slide. Figure 4d,g present the MPM SHG images obtained before and after the sample-stage movement. The SHG images were stacked images with a depth range of 70 µm within the represented orange and blue rectangular boxes in the OCM volumetric image, and this is done to better match and correlate the MPM *en face* images with the OCM *en face* images. The hollow crosses in the red, orange, and blue regions indicated correlates to matched positions in the respective OCM and MPM *en face* images.

### Employing the three-axes positioning of MPM focus point in a biological large-volume sample with guidance of PD–PT OCM

**Figure 5:**
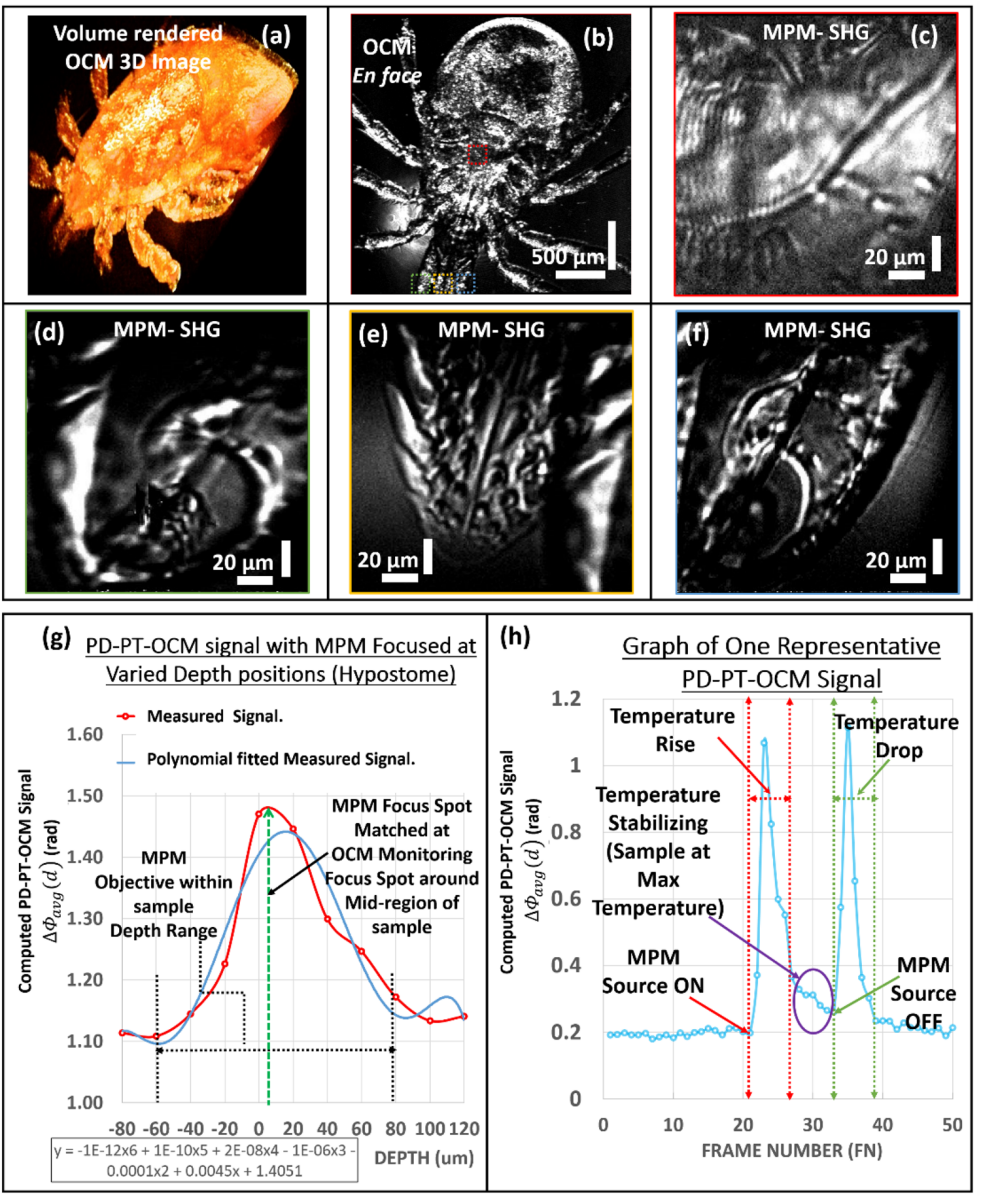
Verification of applicability of the proposed PD–PT OCM-guided MPM for a biological sample. (**a**, **b**) are the respective OCM volume-rendered image and *en face* image of the insect sample. Figures (**c** to **f**) are the MPM *en face* images of different regions in the sample obtained using the PD–PT OCM guidance. (**g**) is the representative photothermal response observed by varying depth position of MPM objective in the hypostome region of the sample, and (**h**) is the representative graph of one PD–PT OCM photothermal response signal.

To validate the usefulness of PD–PT OCM guidance to ROIs in a biological large-volume sample for MPM imaging, a biological specimen (female *Ixodes dammini*) embedded in a microscope slide was used. Prior to photothermal imaging, the specimen was imaged with the maximum FOV of the OCM and post-processed using a volume-rendering software. A volume rendered OCM 3D image is shown in Fig. 5a, and an *en face* image of the volume image at a depth of 65 µm is shown in Fig. 5b. Using the real-time cross-sectional and *en face* OCM images, multiple different sections of the biological specimen were targeted using the ROI selection mechanism based on the photothermal response measurement with the PD–PT OCM. A total area 4 × 4 mm (Vertical × horizontal) is used for ROI selection. In particular, four regions were selected and targeted, which were located at the uppermost surface of the Scutum/shield, top edge of the left palp, tip of the hypostome, and top edge of the right palp. Among these parts, the hypostome, left and right edge of palps were chosen as these parts of the ticks are more useful in studies of tick feeding habitats relating to Lyme disease^53^, and scutum covers the superior portion of the dorsal surface. Almost all parts of the specimen’s body offered a good observable PD–PT-OCM signal, and it is noteworthy that the photothermal signal response reduces with higher thickness. This may be due to the composition and optical and thermal properties of the specimen. The SHG images of the targeted regions are shown in Fig. 5c-f, respectively. The total readings of the observed photothermal responses in the hypostome region according to the depth-varying MPM objective focal positions are shown in the graph plotted in Fig. 5g. The step interval between two depth positions used here is 20 µm. A total of 11 readings were used for covering the entire hypostome region. As can be seen from the graph of a representative photothermal response, as shown in Fig. 5h, the detected maximum phase-differentiated peaks were not inversely proportional. This can be attributed to the thermoelastic expansion of the biological structures of the specimen.

## Discussion

The use of MPM has progressively gained in popularity in diverse biomedical imaging scenarios. One such traction where MPM has grown in applicability is biochip-based research investigations. Biochip-based research is currently evolving from two-dimensional (2D) to 3D platforms, similar to the in vivo microenvironment. In particular, it is necessary to establish large-scale biological complexity to create artificial organs for future drug screening or eventual organ replacements. To achieve this, research is currently being conducted on 3D printing and organoid-based self-organization methods^54,55^. For the long-term live imaging and multiscale analysis of the large-scale specimens, nonlinear microscopy capable of deep tissue imaging and label-free imaging is becoming increasingly important^56,57^. When conducting nonlinear microscopy, care must be taken to avoid photodamage. The proposed method uses endogenous particles within the sample for ROI location in 3D in label-free multiphoton imaging. The proposed method can minimize photodamage by accurately identifying an ROI, where high-resolution 3D molecular imaging is required (supplementary information). This concept is similar to that of phase-contrast microscopy, which is commonly used in fluorescence microscopes to minimize photobleaching, but these conventional guiding mechanism offers only a 2D topological image of sample. OCT can be used as a modality for guided imaging of large-scale, non-transparent, and thick biological tissues in 3D, just as phase-contrast microscopy which is used as a guide imaging tool for transparent specimens.

In the current study, we obtained the photothermal response curve while manually changing the focal point of the MPM objective which is moved towards the observation point (OCM objective). The photothermal response curve has the form of a unimodal function with one maximum value in the measurement area. In future, by incorporating a well-established efficient search method such as the golden-section search^58^, Fibonacci search^59^, or curve-fitting search^60^ (with an automated axial focus motor incorporated with objective lens) instead of a linear search of 50 attempts at intervals of 10 µm; the desired point can be found with the same accuracy in less than 10 attempts. In addition, the search time can be significantly reduced when the high-speed search algorithm is combined with a high-speed motorized device for axial scanning of the focal plane of the MPM objective. In this study, ROI locating in a relatively large biological sample with dimensions of 4 × 4 mm (Vertical × horizontal), a total of 11 readings with 20 µm step interval in depth direction was used. The step interval and total readings can be adaptively chosen depending on the sample composition and thickness.

The probability of error occurrence for ROI location (in depth) for the MPM objective guidance depends on the axial resolution of the OCM system, and the phase sensitivity. The axial resolution of the used OCM system is ~ 5 µm. Hence, the accuracy of MPM objective guidance is same with axial resolution of the OCM. The proposed multimodal imaging system is configured in a way that the OCM and MPM is co-axially aligned and in opposite sides of the sample. The demonstrated method measures the absolute position of the hotspot of the MPM objective within the sample, hence even if a sample with complex structures and sample interfaces is used the performance of the PD–PT-OCM detection is not subject to any erroneous measurements. The FOV of the PD–PT-OCM system can be adaptively changed as per imaging requirements by changing the objective lenses in the sample and reference arm setup. However, the FOV and the lateral resolution are inversely related. In general, wider FOV objectives will have lower lateral resolution and vice versa. The depth-resolved imaging capability of OCM reduces in thick samples. This is due to the limitations of light penetration in thick tissues. Also, in thick samples with densely packed complex structures, the detectable multiphoton generated signals reduce in deeper tissues. Thus the heat generated by the hotspot of MPM will also degrade in deeper tissues. The usability of proposed methods for ROI selection using OCM is not just limited with the phantom sample thickness reported here. The ROI selection within the sample is possible along the entire optical depth range of OCM and is only dependent on sample composition and the optics used for the OCM system. The photothermal signal generated within the sample can be effectively detected along the entire observable depth range of the PD–PT-OCM. The photothermal signal dissipation and degradation of detection efficiency will depend on the sample thickness, composition, and its optical and thermal properties^51^.

Conventionally, photothermal OCT imaging is used to image blood vessels using the photothermal effect of blood cells, which are endogenous substances, or to measure the location and hot spots of exogenous photoreactive particles such as polymers and gold nanorods in living tissues. The highly sensitive PD–PT OCM can also be used if endogenous or exogenous factors are present within the observable range of the OCM image. Using the proposed method, we can carefully target the excitation illumination for photothermal therapy with exogenous substances and locally restrict it to the desired location, thus avoiding unnecessary heating of normal tissues.

In summary, this paper serves as a preliminary report to present the potential usability of employing PD–PT OCM as a guiding tool for nonlinear microscopy to effectively and accurately navigate ROIs in a large-volume samples. The ROIs were selected by utilizing the large FOV and observable depth range in *en face* and cross-sectional OCM images. To achieve targeted MPM imaging within the ROIs in 3D, hotspots occurring within the sample focused with the MPM laser at a weak power were detected using the highly sensitive PD–PT OCM. We then focused the MPM laser on an observation point selected from the endogenous absorption agents in the ROI. The PD–PT OCM allows the focal position of the MPM objective to be placed on the ROI within the observable depth range of the OCM cross-sectional image. Effectively finding and targeting ROIs requiring high-resolution MPM imaging can reduce the overall exposure time of high-power lasers, especially for large samples, significantly mitigating photobleaching, and photodamage. The high-sensitivity PD–PT OCM system has potential applications for photothermal reaction analysis because it can detect in real-time even temperature changes due to weak photothermal perturbation occurring within three-dimensional biological samples. Furthermore, it is to be noted that the utility of the proposed PD–PT OCM-based guiding method can be implemented for any nonlinear imaging technique that requires high-power laser illumination (which may lead to photobleaching and photodamage in samples) such as two-photon excited fluorescence and coherent anti-Stokes Raman scattering microscopy. This reduces the overall acquisition time required for the ROI location in nonlinear imaging systems.

## Materials and methods

### Multimodal imaging system design and specifications

**Figure 6:**
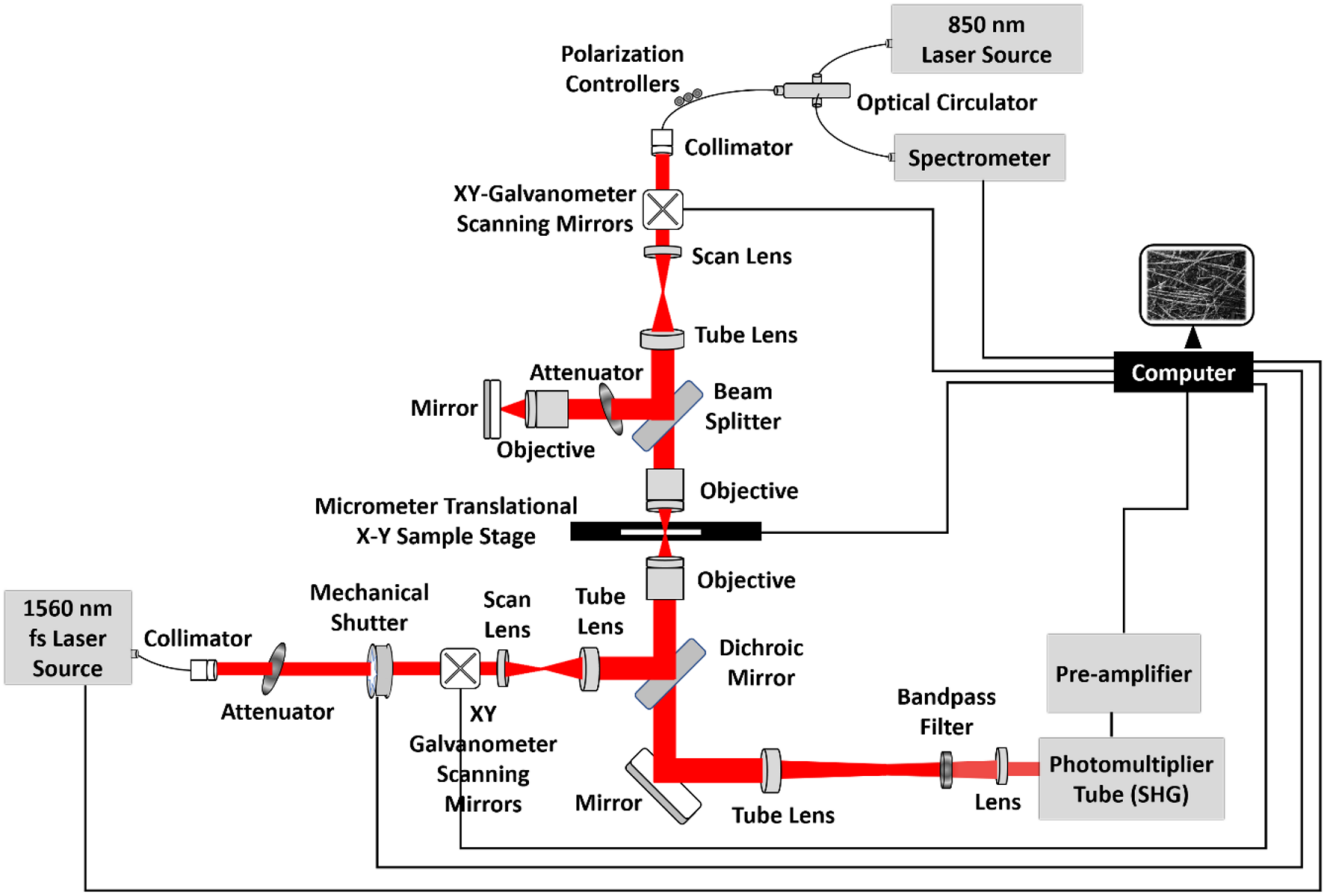
Schematic of the multimodal imaging system. Optical design schematic with the optical components used along with the MPM and OCM sources, detection components, and two-axes micrometer translational sample stage. Figure not drawn to scale.

The overall configuration of the multimodal imaging system is illustrated in Fig. 6. The multimodal imaging platform had two independent light sources. The OCM system was powered by a super-luminescent broadband light source (SLD-37-HP3, Superlum Diodes Ltd., Carrigtwohill, Ireland) with a maximum power output of 18 mW centered at 840 nm. The output from the source was directly connected to the entrance port of an optical circulator (850-H7-L-15-FA, OF-LINK Communications Co., Ltd. Shenzhen China). The output port of the circulator was connected to a doublet collimator (F240APC-850, Thorlabs, Inc., NJ, USA) with a beam size 2.4 mm. The collimated beam was raster-scanned along the horizontal and vertical axes using a dual-axis galvanometer scanning mirror (GVSM002-JP, Thorlabs, Inc., NJ, USA). Subsequently, the scanning beam was directed towards a combination of scan and tube lenses with focal lengths of 54 mm (LSM04-BB, Thorlabs, Inc., NJ, USA) and 200 mm (TTL200-B, Thorlabs, Inc., NJ, USA), respectively. With this configuration, the optical beam size (from the collimator) was magnified by a factor of 3.7. The enlarged beam was then split by a rectangular beam splitter (BSW11, Thorlabs, Inc., NJ, USA) at a 50:50 ratio. The split beams were directed to the reference arm and the sample arm setup. The beam towards the reference path passed through a combination of optical systems such as a continuous variable attenuator (NDC-50C-4 M, Thorlabs, Inc., NJ, USA) and a 4 × objective lens (UPlanFL N 4.0x, Olympus, Tokyo, Japan) with a focal length of 45 mm, followed by a high-reflective broadband mirror (PF10-03-P01P, Thorlabs, Inc., NJ, USA). Similarly, in the sample path, the incident split beam was directed to an objective lens that matched the specifications of the objective lens used in the reference path. The back-reflected laser beam from the sample surface interfered with the back-reflected reference beam in the beam splitter. The backscattered interference signal was directed via a circulator to a spectrometer containing a line-scan camera (Sprint spl4096-140k, Basler AG, Ahrensburg, Germany) with 4096 pixels. In the current configuration, the spectrum covered only the middle part of the camera sensor, and only 2048 pixels in the line scan camera were used. The detected signals were processed on a computer to display the cross-sectional and *en face* OCM images in real-time. The built OCM system had axial and lateral resolutions of ~ 5 µm, and a maximum FOV of 4 × 4 × 1 mm along the horizontal, vertical, and depth axes, respectively. A set of 250 lateral positions (A-line) was scanned successively to construct one 2D cross-sectional OCM image, and 250 horizontal positions were scanned (2D images) to obtain one volume image set. A specific depth was selected from the volume image set (as required) to obtain an *en face* image in real-time. The OCM system had an average speed of 90 frames per second.

The MPM system had a high-power femtosecond fiber laser source (ELMO HP, Menlo Systems, Inc., NJ, USA) with a maximum output power of 180 mW, and a pulse width of < 70 fs (typically 45 fs), and a repetition rate of 100 MHz. The laser source was centered at 1560 nm, with a spectral bandwidth of 30 nm. The laser beam was collimated using an optical triplet collimator (TC18APC-1550, Thorlabs, Inc., NJ, USA) with a beam size of approximately ~ 3 mm. The laser power was controlled as required using a combination of optical attenuators. The propagating beam then passed through an electronically controlled mechanical shutter (SHB025, Thorlabs, Inc., NJ, USA). The computer controlled the mechanical shutter to obtain optical illumination in the sample region during a specific operation time, as required. The subsequent laser beam after the shutter was raster-scanned along the horizontal and vertical axes using a dual-axis galvanometer scanning mirror (GVSM002-JP, Thorlabs, Inc., NJ, USA). The laser beam was then passed through a combination of scan lens (SL50-2P2, Thorlabs, Inc., NJ, USA) and tube lenses (TTL200MP, Thorlabs, Inc., NJ, USA) with focal lengths of 50 mm and 200 mm, respectively. This optical combination magnified the laser beam by a factor of 4. The collimated and magnified laser beam propagated through a dichroic mirror (FF801-Di02-25 × 36, IDEX Health & Science, LLC, New York, USA). The dichroic mirror was placed at an inclination angle of 45° with respect to the incident laser beam. The redirected MPM laser beam was then focused on the sample using a 30 × aspheric objective lens (5723-C-H 30x, Newport Corporation, CA, USA) with a focal length of 6.2 mm. The focused MPM laser beam on the sample generated the SHG signals from molecules of samples, which were backscattered and collected by the objective lens. The backscattered SHG signals were then transmitted through the dichroic mirror, which was directed to a tube lens (TTL200MP, Thorlabs, Inc., NJ, USA) with a focal length of 200 mm, bandpass filter (FF01-794/32-25, IDEX Health & Science, LLC, New York, USA), and achromatic doublet lens (AC254-050-B, Thorlabs, Inc., NJ, USA) with a focal length of 50 mm. This beam was transmitted to a photomultiplier tube (H7422-50, Hamamatsu Photonics, Shizuoka, Japan). The electrically converted signals were pre-amplified and then transmitted to a computer for further processing, to obtain the sample's *en face* MPM SHG images. The MPM system had a resolution of approximately ~ 1 µm. With the stated optical configuration, a maximum FOV of 300 × 300 µm was achieved.

### The ROI selection from the OCM image and MPM targeting on the ROI

The OCM and MPM imaging systems were mounted onto a microscope (OLYMPUS-IX73, Olympus, Tokyo, Japan) from the top and bottom directions, respectively, and the OCM and MPM laser beams were coaxially aligned. The x- and y-coordinates of the ROI for the high-resolution MPM were selected from the real-time *en face* OCM image. We selected the depth coordinate of the ROI from the correlated cross-sectional OCM image, which was the depth position for observing the photothermal response. The lateral targeting of the selected ROI for the MPM was implemented by the translational movement of a micro-positioning (X and Y) stage (MCL-MOTNZ, Mad City Labs Inc., WI, USA). The MPM excitation light was then focused on the depth position of the ROI by observing the changes in the photothermal response while changing the focal position of the MPM with respect to the in-built axial motion of the microscope body. The sample translational movement was automated using the LabVIEW program, whereas the axial movement was controlled manually.

### Simple and complex network phantom sample fabrication and experimental protocol

**Figure 7:**
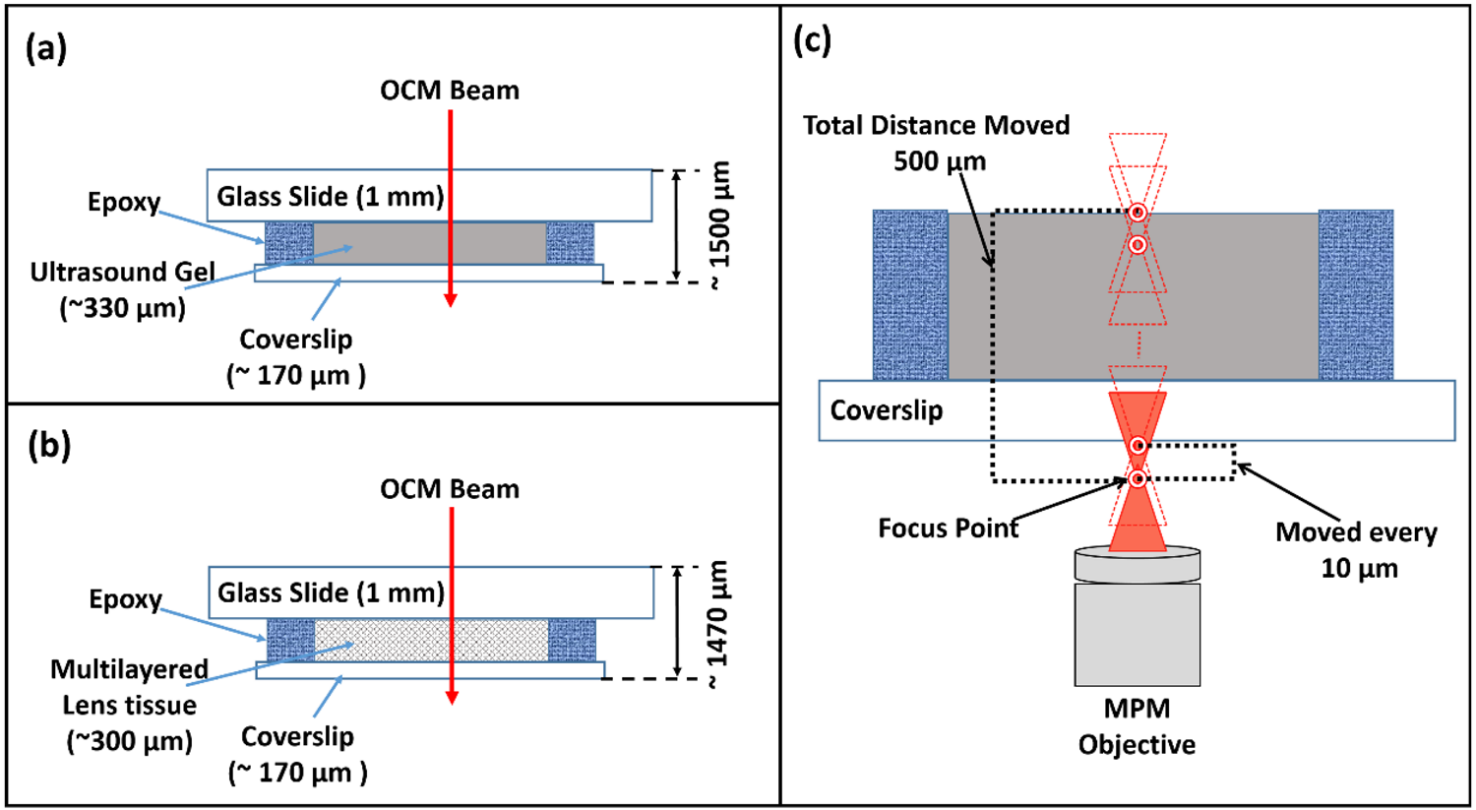
Schematic of phantom samples and the PD–PT OCM experimental protocol. (**a**) is the schematic cross-sectional representation of the simple phantom sample fabricated with ultrasound gel. (**b**) is a schematic cross-sectional representation of the complex network phantom sample fabricated with multilayered lens tissue. **c** is the figurative depiction of MPM objective positioning for the PD–PT OCM based methodology. Figures not drawn to scale.

To evaluate and analyze the methodology and robustness of the proposed multimodal PD–PT OCM-guided MPM imaging system, two phantom samples were fabricated. One of the samples was prepared by placing a commercially available ultrasonic gel on a 1 mm glass slide, and a 170 ± 5 µm thick coverslip was stacked on the ultrasonic gel, as shown in Fig. 7a. Here ultrasonic gel is used as its thermal properties are more stable and it thermal conductivity is close to biological tissues^61^. The overall thickness of the fabricated sample was ~ 1500 µm, and the thickness of the introduced ultrasound gel was ~ 330 µm. Similarly, we developed a multilayered complex network phantom sample to mimic complex network structures in biological tissues, as shown in Fig. 7b. Multilayered lens tissues with an overall thickness of ~ 300 µm and distilled water were used instead of the ultrasound gel, and the total sample thickness was ~ 1470 µm. A rapid set epoxy was used to seal the area surrounding the coverslip in both samples to prevent the evaporation of water. The sample was mounted with a coverslip oriented towards the MPM objective, while the glass slide was oriented towards the OCM, as shown in Fig. 7a,b. To demonstrate the usefulness of PD–PT OCM guidance to ROIs in a biological large-volume sample for MPM imaging, a biological specimen (*Ixodes dammini* (Deer Tick) Female, w.m. Microscope Slide, Carolina Biological Supply, NC, USA) embedded in a microscope slide was used which was 3 mm in length and width (excluding some parts of legs). To obtain the PD–PT OCM photothermal responses of the phantom samples and the biological specimen, the initial MPM objective focus position was placed 30 µm below the coverslip, and the MPM objective lens was moved axially towards the sample in increments of 10 µm step interval, as shown in Fig. 7c. Upon each movement of the MPM objective, the mechanical shutter was set to open for the desired time period (350 ms, unless mentioned otherwise) for each PD–PT OCM signal acquisition. The observation points of the OCM, where the photothermal responses were measured, were selected from the points within the ROI that provided the appropriate photothermal signal. Principally, the step interval specified here is not an absolute value that must be strictly adhered to. For instance, a 20 µm step interval was employed to generate the photothermal response of the biological specimen (female *Ixodes dammini*) depicted in Fig. 5. The step interval and total readings can be selected adaptively based on the composition and thickness of the sample.

## Supplementary Information

Supplementary Information.

## Data Availability

All the necessary data required to analyze and reproduce the results and conclusions presented in the manuscript are provided in relevant sections in the manuscript. Additional details and data related to this study are available from the corresponding author upon reasonable request.
