# Supplementary material for: Label-free photothermal optical coherence microscopy to locate desired regions of interest in multiphoton imaging of volumetric specimens
Source: Sci Rep. 2023 Mar 3;13:3625. doi: 10.1038/s41598-023-30524-z (PMC9984493; doi:10.1038/s41598-023-30524-z)
Supplement: Supplementary file 1 — Supplementary Information. [file 41598_2023_30524_MOESM1_ESM.pdf]

Supplementary File:
Label-free photothermal optical coherence microscopy to locate desired regions of interest in multiphoton imaging of volumetric specimens

Naresh Kumar Ravichandran[1], Hwan Hur[1], Hyemi Kim[1], Sangwon Hyun[1], Ji Yong Bae[1], Dong Uk Kim[1], I. Jong Kim[1], Ki-Hwan Nam[1], Ki Soo Chang[1]*, Kye-Sung Lee[1]*

[1]Center for Scientific Instrumentation, Korea Basic Science Institute, 169-148 Gwahak-ro Yuseong-gu, Daejeon 34133, Republic of Korea.

Sample Description:

A 3D biochip sample was used to demonstrate the usefulness of the proposed label-free PD-PT-OCM for MPM imaging with reduced photodamage during ROI positioning. The 3D biochip sample contains breast cancer spheroid surrounded by vasculature which is made of breast cancer cells (MDA-MB-231), fibroblast (NHLF), and endothelial cells (HUVEC). First, breast cancer spheroids were formed by culturing a mixture of MDA-MB-231 and NHLF cells with a 6:1 ratio in PrimeSurface® 3D culture plate for 3 days. Then, the breast cancer spheroids, NHLF cells, and HUVECs were mixed with a complete culture medium containing 4 units of thrombin and 6 mg/ml fibrinogen and injected into a microfluidic chip to form vasculature. The cells in a microfluidic chip were cultured for 6 days with an EGM-2 medium containing 50 ng/ml of VEGF-A. For long-term preservation, the 3D biochip sample was fixed with 4% paraformaldehyde and filled with a mounting medium. The sample consisted of multiple spheroids, of which two regions were selected as ROIs for the experiment. Fig. S1 and Fig. S2 are the MPM images of the ROI-1 and ROI-2 of the spheroids grown in the biochip sample.

Result:

To simulate the conventional image-based ROI search method the sample was imaged using MPM at ten different depth positions spaced 10 μm apart within the spheroid region. This is considered as one process of searching and imaging (ten x-y images at different depths) in the sample. The process was then repeated ten times to simulate repeated observations likely in practical experiments. The incident power on the sample for the process of searching and imaging was about 75 mW. The high incident power was used to maximize the photodamage in a short time. In Fig. S1 the images (A) to (J) are the MPM x-y images taken at the same depth position (ROI-1) from each of the ten sets acquired sequentially. (K) and (L) are the enlarged views of images (A) and (J) respectively, and (M) is the structural differences between (K) and (L). Images (N) and (O) are the x-y images of sets one and ten taken at 50 μm above the ROI-1 depth, and (P) is the structural differences between (N) and (O). It took 36 seconds for each x-y imaging. As

indicated by the red arrows in Fig. S1, it can be seen that the hole appears around the sixth process and continues to grow with the number of processing.

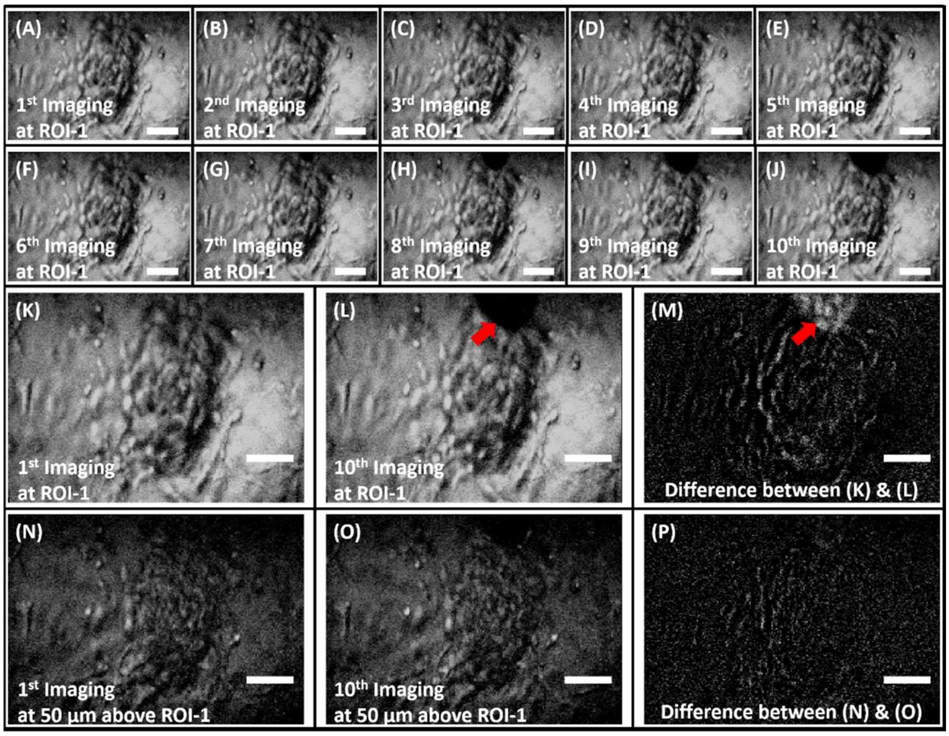

Fig. S1. MPM imaging of 3D biochip sample with conventional full power image-based ROI locating along depth. (A)-(J) are series of MPM x-y images of ROI-1 from the first to the tenth sets acquired during the ROI search processes. (K) and (L) are enlarged images of (A) and (J) respectively. (M) shows the structural differences between (K) and (L). (N) and (O) are the first and the tenth MPM x-y imaging acquired at 50 μm above the ROI-1 depth, and (P) shows the structural differences between (N) and (O). Scale bars are 100 μm.

To evaluate the merits of the proposed method, the photothermal OCT-based ROI search method was repeated 10 times as above. The ROI searching was done in the same way as explained in the sub-section of the materials and methods translating the MPM objective along the depth axis at intervals of 10 μm. The MPM x-y imaging was then done at the selected depth of ROI-2. The MPM incident power during the ROI-2 search was about 4 mW. On the other hand, the laser power for imaging was about 75 mW which is the same as in the conventional image-based ROI search method. Images (A) to (J) in Fig. S2 are the MPM x-y images taken at the same depth position (ROI-2) from each of the ten sets acquired sequentially. Images (K) and (L) are enlarged views of sets one and ten respectively, whereas (M) depicts the structural changes between (K) and (L). (N) and (O) are the x-y images of sets one and ten taken 50 μm above ROI-2 depth, and (P) is the structural differences between (N) and (O).

During the image-based ROI locating process, the sample was focused with high power intensity for imaging every 10 μm depth, resulting in the structural changes that appeared to be caused by photodamage at all depths, as shown in the images (M) and (P) of Fig. S1. On the other

hand, optical damage could be reduced significantly not only at the ROI depth but also at other depths, which can be seen in (M) and (P) of Fig. S2. This is owing to the advantages of having reduced overall light exposure and imaging numbers required with the proposed PD-PT-OCM-based ROI search method.

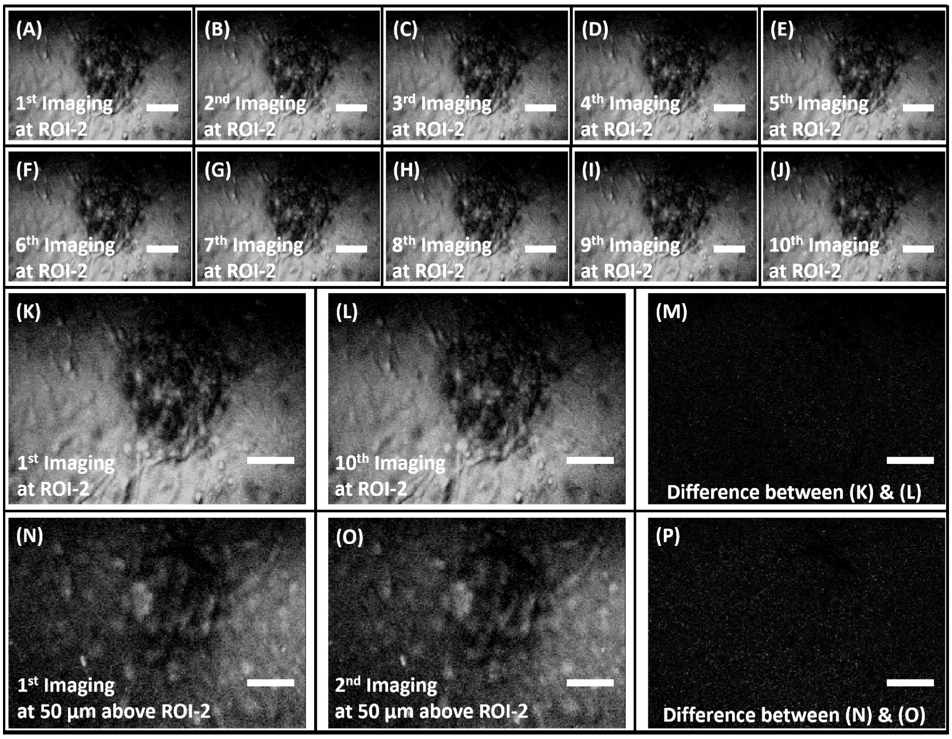

Fig. S2. MPM imaging of 3D biochip sample with PD-PT-OCM guided ROI locating along depth. (A)-(J) are series of MPM x-y images of ROI-2 from the first to the tenth sets acquired during ROI search process. (K) and (L) are enlarged images of (A) and (J), and (M) is the difference between (K) and (L). Images (N) and (O) are the first and second MPM x-y imaging acquired at 50 μm above the ROI-2, and (P) is the difference between (N) and (O). Scale bars are 100 μm.
